# Cancer clinical trial participation: a qualitative study of Black/African American communities’ and patient/survivors’ recommendations

**DOI:** 10.1093/jncics/pkae119

**Published:** 2024-11-25

**Authors:** Linda Kaljee, Sylvester Antwi, Doreen Dankerlui, Donna Harris, Barbara Israel, Denise White-Perkins, Valerie Ofori Aboah, Livingstone Aduse-Poku, Harriet Larrious-Lartey, Barbara Brush, Chris Coombe, La’Toshia Patman, Nayomi Cawthorne, Sophia Chue, Zachary Rowe, Cassandra Mills, Kurt Fernando, Gwendolyn Daniels, Eleanor M Walker, Evelyn Jiagge

**Affiliations:** Henry Ford Health, Global Health Initiative, Detroit, MI 48202, United States; Henry Ford Health, Detroit, MI 48202, United States; Henry Ford Health, Global Health Initiative, Detroit, MI 48202, United States; Grace Learning Center, Detroit, MI 48228, United States; University of Michigan Detroit Urban Research Center, Ann Arbor, MI 48109, United States; Department of Family Medicine, Henry Ford Health, Detroit, MI 49224, United States; Henry Ford Health, Detroit, MI 48202, United States; University of Florida, Gainesville, FL 32611, United States; Henry Ford Health, Detroit, MI 48202, United States; University of Michigan Detroit Urban Research Center, Ann Arbor, MI 48109, United States; University of Michigan Detroit Urban Research Center, Ann Arbor, MI 48109, United States; Our Wellness Hub, Detroit, MI 48214, United States; Eastside Community Network, Detroit, MI 48215, United States; Caribbean Community Service Center, Detroit, MI 48224, United States; Friends of Parkside, Detroit, MI 48213, United States; Henry Ford Health, Detroit, MI 48202, United States; Henry Ford Health, Detroit, MI 48202, United States; Institute for Population Health, Detroit, MI 48202, United States; Henry Ford Health, Detroit, MI 48202, United States; Henry Ford Health, Detroit, MI 48202, United States

## Abstract

**Background:**

Black/African Americans experience disproportionate cancer burden and mortality rates. Racial and ethnic variation in cancer burden reflects systemic and health-care inequities, cancer risk factors, and heredity and genomic diversity. Multiple systemic, sociocultural, economic, and individual factors also contribute to disproportionately low Black/African American participation in cancer clinical trials.

**Methods:**

The Participatory Action for Access to Clinical Trials project used a community-based participatory research approach inclusive of Black/African American community-based organizations, Henry Ford Health, and the University of Michigan Urban Research Center. The project aims were to understand Black/African Americans’ behavioral intentions to participate in cancer clinical trials and to obtain recommendations for improving participation. Audio-recorded focus group data were transcribed and coded, and searches were conducted to identify themes and subthemes. Representative text was extracted from the transcripts.

**Results:**

Six community focus group discussions (70 participants) and 6 Henry Ford Health patient/survivor focus group discussions (29 participants) were completed. General themes related to trial participation were identified, including (1) systemic issues related to racism, health disparities, and trust in government, health systems, and clinical research; (2) firsthand experiences with health care and health systems; (3) perceived and experienced advantages and disadvantages of clinical trial participation; and (4) recruitment procedures and personal decision-making processes. Specific recommendations on how to address barriers were obtained.

**Conclusions:**

Community-based participatory research is effective in bringing communities equitably to the table. To build trust, health systems must provide opportunities for patients and communities to jointly identify factors affecting cancer clinical trial participation, implement recommendations, and address health disparities.

## Introduction

In the United States, Black/African Americans have a disproportionate cancer burden, including the highest mortality (overall number of deaths) and the lowest survival rates (how long people live after their diagnosis) of any racial or ethnic group for most cancers.^1^ Among Black/African American men, overall cancer incidence is 6% higher than among White men, and mortality is 19% higher. Black men are at 2 times higher risk of death from myeloma, stomach cancer, and prostate cancer. Black/African American women have an 8% lower cancer incidence than White women but a 12% higher mortality rate. Black/African American women are 2 times as likely to die from endometrial cancer and 41% more likely to die from breast cancer^1^ than White women (cancer-specific mortality). Racial and ethnic variation in cancer burden reflects health inequities, disparate recommendations among health-care professionals, variations in risk factors for cancers, and heredity and genomic diversity within and across ethnic groups.^2-6^ In addition, Black/African Americans are less likely to have access to and participate in cancer prevention, screening, treatment, and clinical trials.^6-8^

Black/African Americans account for more than 12% of the population in the United States, but on average, clinical trials include only about 5% Black/African American participants.^9^ Barriers to participation in cancer clinical trials affect data integrity and contribute to poor outcomes for Black/African Americans because their tumors are not equally represented in new drug discovery efforts.^1^ Multilevel factors contribute to low participation. Societal factors include historical trauma and experiences with systemic racism^8^^,^^10^ and eroding trustworthiness of government, pharmaceutical companies, and health systems. Health systems and research teams may fail to address communication barriers, use plain language to describe trials,^11^^,^^12^ provide accessible information about trials,^13^ and help patients navigate complicated trial logistics and social and economic impact.^14^ In addition, limited community outreach efforts on the part of health systems, health-care professionals’ implicit biases, and lack of diversity among clinicians to meet patient preferences for ethnic concordance are also important contributors.^15-17^

Henry Ford Health (HFH) serves a diverse population in Detroit, Michigan, and the larger southeastern Michigan region. The Henry Ford Cancer Institute (HFCI) is 1 of 20 sites that offer clinical trial services in the Michigan Cancer Consortium.^18^ In response to disparities in both cancer outcomes and participation in cancer clinical trials between Black/African American and White patients, the first phase of the Participatory Action for Access to Clinical Trials (PAACT 1.0) project was implemented in 2021. PAACT 1.0 used a community-based participatory research approach to ensure a collaborative partnership with Detroit-based Black/African American community organizations. The overall goals of PAACT 1.0 were to

- increase knowledge about Black/African American community members’ and cancer survivors’ behavioral intentions to participate in cancer clinical research trials and the determinants affecting their access to trials and decisions regarding participation;
- increase knowledge about HFCI clinicians’ perceptions of why Black/African American patients are not participating in clinical trials and changes in institutional, clinical, and trial processes and procedures that could facilitate Black/African American participation; and
- obtain recommendations to develop or adapt strategies and interventions at the institutional, clinical, and community levels to support an increase in Black/African American cancer clinical trial participation.

This article focuses on qualitative focus group data with Black/African American community members and HFH patients with cancer and cancer survivors to elucidate barriers to trial participation and community and patient/survivor recommendations to address those barriers. Between 2023 and 2026 (PAACT 2.0), these data will be used to pilot and evaluate recommended intervention strategies through Black/African American community-based organizations, within HFCI clinics, and at the HFH system level.

## Methods

### Community-based participatory research

Community-based participatory research strives for equitable involvement of community members, organizational and health system representatives, public health and other practitioners, and researchers in all aspects of the research process.^19^^,^^20^ Community-based participatory research has been shown to be a positive and effective approach in relation to clinical trial participation among diverse populations.^21^^,^^22^ Outcomes have included identification of successful interventions as well as increases in recruitment and retention of targeted populations. To a lesser extent, these studies have also included descriptions of the role of community members in data interpretation and dissemination. In this project, community members and patients/survivors were involved in all aspects of the development of data-collection tools, data review, interpretation and dissemination, and identification of key themes and recommendations. In PAACT 2.0, community members will be engaged in the selection of recommendations to be piloted and evaluated and ongoing review of outcomes from the process of implementing interventions at the community, clinical, and system levels.

Community-based participatory research partnerships embrace principles of mutual respect, power sharing, co-learning, balancing research and action, and a long-term commitment to achieving racial and health equity.^20^^,^^23^^,^^24^ The PAACT community-based participatory research partnership engaged and expanded on the community-based participatory research principles established by the Detroit Urban Research Center.^25^^,^^26^ PAACT established a steering committee with representatives from 8 community-based partners, 4 researchers from HFH, and 2 researchers from the University of Michigan.

The study was designed to include diverse representation of Black/African Americans living in the Detroit metropolitan area and included African American, Caribbean, and Ghanaian communities. The term *Black* can be used to be inclusive of all people of African descent within the United States. After discussions among Black/African American research team members and the steering committee, the decision was made to use both terms to be inclusive of (1) participants who represent diasporan Africans born in the United States and more recent immigrants from Africa and the Caribbean and (2) diasporan individuals born in the United States who vary in how they self-identify as Black or African American.

### Study site

The study was conducted through the HFCI and the HFH Global Health Initiative in partnership with the University of Michigan Schools of Nursing and Public Health and the Detroit Urban Research Center.

### Participant recruitment

Research participants included community members from our partner organizations and patients/survivors from HFCI with a diagnosis of breast, colon, lung, or prostate cancer. The HFCI patients/survivors were identified through a search within the electronic health records system. Filters included patient identification as Black/African American and type of cancer diagnosed. Dates of inclusion were January 1, 2019, to December 31, 2020. Eligible patients were contacted by email by PAACT staff with an invitation to participate in a virtual focus group. When patients agreed to participate, they were sent a link to a Research Electronic Data Capture consent form and an optional demographic questionnaire. Although all the patient/survivor focus groups were virtual, participants had the option to sign up by telephone and have a paper consent form mailed to them. None of the participants opted for the latter recruitment and consenting strategy.

Community members were identified and contacted by the steering committee members, who represented the community organizations. Community member focus groups were organized either in person or virtually. In-person community focus group participants completed consent forms immediately before data collection. Virtual community focus groups followed the consenting procedures described earlier. This study was approved by HFH Institutional Review Board Committee.

### Data collection, management, and analysis

The qualitative data-collection, management, and analysis work was led by Linda Kaljee, PhD. Dr Kaljee is trained in anthropology and has more than 30 years of work experience both in community participatory research and in qualitative data methodologies. Doreen Dankerlui, Sylvester Antwi, and Evelyn Jiagge, MD, PhD, were also involved in data collection and have experience in qualitative data methodology. The analysis and interpretation of the data included the full research team and the PAACT Steering Committee. Interview guides were developed in partnership with the PAACT Steering Committee for each population (patients, community members). Guides were developed based on a comprehensive literature review focused on barriers and facilitators for Black/African American participation in cancer clinical trials at the social-historical, institutional, clinical, community, and personal levels^27^ (see Supplementary Methods, available online). In addition, respondents were asked to provide specific recommendations to address issues that affect Black/African American clinical trial participation. Focus group discussions were audio-recorded and transcribed. Notes were also taken during the interviews to identify new and emerging data. Transcribed data were uploaded to a web-based qualitative data management program (Dedoose). Dedoose provides simultaneous access to the data by multiple team members.

A content analysis approach was used and included an interpretive, naturalistic approach focused on the narrative data. The focus groups were approximately 1 hour in length. Dr Kaljee and 3 other research team members developed the coding dictionary. Intercoder reliability was established through team members’ independent coding of transcripts and a review of those coded texts. The data analysis team held regular meetings during coding, coding searches, and data analysis and interpretation. Themes were identified through the literature review and the emergence of information during data analysis. Analyzed data were separated into tables by codes and identified themes within the coded data. These tables were shared with the broader research team and the steering committee members to review, and themes were modified based on multiple perspectives. Final data tables included codes, themes, and representative text from the transcripts.

### Community forums

Following the community-based participatory research approach, 2 community forums were organized after initial data analysis to provide an opportunity for patients and community members to review and interpret outcomes and recommendations. Participants also determined the most appropriate and relevant recommendations to address barriers to participation in cancer clinical trials. Using an iterative process, participants prioritized the recommendations based on impact and feasibility.

Community-based members of the steering committee served as recruitment ambassadors, and they identified community members who were interested in attending the community forums. Tools fostering education, trust building, and sustainability guided the community forums. Media communication was clearly written and relevant to Black/African American communities. Most importantly, the forums were designed to address why community voices are essential to increasing equitable engagement in cancer clinical trials and cancer care.

The community forums included a total of 83 participants. Accommodations with transportation, language interpretation, food, and incentives were provided for the participants.

### Ethical approval

The study was approved by the HFH Institutional Review Board (study title: “Participatory Action for Access to Clinical Trials”; study No. 14889; principal investigator: Dr Evelyn Jiagge). All study participants signed written consent forms before data collection.

## Results

Six community focus group discussions with 70 participants and 6 HFCI patient focus group discussions with 29 participants were completed. The research team was confident that the final sample size reached saturation in terms of identification of barriers and facilitators to participation in cancer clinical trials and recommendations to address those barriers. Community focus groups included 1 group each of Ghanian and Black Caribbean individuals and 4 groups from community-based organizations in various locations throughout Detroit. Among the survivor groups, 69% (20/29) had been diagnosed with breast cancer, 17% (5/29) with prostate cancer, 10% (3/29) with lung cancer, and 3% (1) with colon cancer.

General themes were identified that contributed to decisions to participate or not in cancer clinical trials: (1) system issues related to racism, health disparities, and trust in government, health systems, and clinical research; (2) personal experiences with health care and health systems; (3) perceived and experienced advantages and disadvantages of participating in clinical trials; and (4) recruitment procedures and personal decision-making processes. As part of the focus group discussions, participants were also asked to share recommendations for addressing the barriers to Black/African Americans’ participation in cancer clinical trials. These recommendations were compiled and presented at the community forums. Illustrative quotes of the themes are included in tables. The recommendations presented are those of the participants, not those of the research team.

### Systemic issues, racism, health disparities, and trust

Issues and concerns related to systemic racism and health disparities were discussed. These deep-seated experiences were both historical and current and related to health care in general, health-care practices, and clinical trials. Participants reported a reluctance to participate in clinical trials because of previous harm and purposeful neglect from government and health systems. They reported that their prior experiences within health-care systems and more general historic and personal experiences with racism strongly affect decisions about participating in clinical trials. In addition, patients’ experiences throughout their cancer diagnosis as well as treatment and care affect feelings of trust and distrust in the system, the health facility, and health-care professionals. Concerns were raised in relation to how patients’ organic samples (eg, tumor cells) are used beyond the immediate required testing for cancer diagnosis, care, and treatment. Another area of concern related to the trustworthiness of information and sources of information regarding clinical trials as well as a lack of information provided to clinical trial participants about trial outcomes and benefits to Black/African American communities.

Respondents discussed the lack of trustworthiness of health systems and health-care professionals. Concerns were raised about disparities in access to health care for Black/African American communities and specifically access to information about preventive care and screenings for cancer (see Table 1).

**Table 1.** Systemic issues, racism, health disparities, and trust

Issue	Illustrative quote
Historical trauma, lack of trust	*“I think historically we as Black people have heard all the horror stories about like the Tuskegee trials where people didn’t get treated like they should, Henrietta Lacks … she didn’t benefit at all nor her family benefited for a long time … . And it’s like in the back of our mind … . Even though you might trust the [clinician] you’ve been dealing with.”* (Community)
Trustworthiness of information and lack of information provided by health systems or clinicians Disparities in access to preventive care or screening information	“*And the thing that upset me about it, after they do the clinical trial, we didn’t get no results back. We didn’t know how this affected their body. Was it positive, was it negative … and I have a big problem with that because that showed lack of transparency.”* (Community)
	“*[W]hen you go to a physician and your gut feeling tells you that this person’s not looking out for your best interest, you don’t want to do anything that they tell you to do.”* (Community)
	“*They [clinicians] don’t tell me ways to prevent things, preventive medicine. It’s more like … fixing a disease … . It’s more like waiting for something to happen and then doing something about it instead of preventing it in the first place.”* (Community)

### Personal experiences with health-care professionals and health systems

Participants discussed positive and negative experiences related to both general health care and cancer-related prevention and care that affect their decision to participate in clinical trials. Communication emerged as a key issue for the development of trusting relationships with health-care professionals and for building confidence in the information received. Participants cited the clinical time that clinicians have with patients as a major contributor to communication of information. Respondents expressed that some health-care professionals were not responsive to their concerns about symptoms, side effects, or use of medications.

Respondents discussed experiences related to cancer prevention, diagnosis, and treatment. They discussed the importance of their clinicians showing interest not only in offering clinical trials after cancer diagnosis but also in information about how they can live healthy lives to prevent disease. Some respondents also felt that how they received their diagnosis was impersonal and did not provide them with an immediate opportunity to discuss the situation with their clinician. Others discussed the lack of information provided to them about treatments or being in situations in which the information provided was overwhelming and confusing. All these factors led to a lack of trust in the clinicians (see Table 2).

**Table 2.** Personal experiences with clinicians and health systems

Issue	Illustrative quote
Positive and negative experiences with health-care professionals	“*Well, with my current primary health provider, I feel like I have a really good relationship with her because she actually listens to my concerns. And when I do go to see her, I don’t feel rushed.”* (Patient)
	“*First of all, I go only [to my primary care physician] when I need to. And when I do go, I feel like it’s on the assembly line. Like he’s in a rush … they have to see so many patients in a very short period of time … . I don’t feel like I get the attention that I should be getting.”* (Community)
Lack of responsiveness from clinicians in relation to addressing symptoms Lack of information about symptoms to expect from cancer treatments	*“And I just feel like it took a long time to find the right provider who could help me manage the symptoms that I was experiencing. I like ended up going to multiple [obstetricians/gynecologists], and that was a very disheartening experience.”* (Patient)
	*“When I first got diagnosed, I had no idea about early menopause. I had no idea about it; you might have to take pills the rest of your life. I had no ideas about all these things … . Somebody should have been explaining all of this to me, so I could have looked at all of my options and decided what’s best for me. And I just felt like I wasn’t given options. And then I had to go home … and do all this research myself.”* (Community)
Lack of information about screenings	*“There are some doctors who don’t even give Black women instructions on when you should be doing a mammogram, or … like colonoscopies and all those early detection screenings.”* (Community)
Diagnosis of cancer over the telephone	*“Well, I had felt a lump, so I had made an appointment to go in and see my doctor and she scheduled some tests. I found out via a phone call at work that it was cancer … . It sucks to find out at work because you’re in shock.”* (Patient)
Too much information and pressure on patients to make decisions	*“They found out that I had cancer of the lung. And so, when they came in to tell me, it was just like, the doctor was nice. He explained everything and [I] understood everything. But after that … it was explaining what they wanted to do … and I said, ‘Whoa, whoa. You just told me this, and the next day you’re telling me all this stuff … that’s really scary.’ But every day, 3 or 4 doctors came in. ‘Did you decide yet? Did you make a decision?’”* (Patient)

### Perceived and experienced advantages and disadvantages of participation in cancer clinical trials

Respondents discussed multiple advantages and disadvantages of participation in clinical trials. In terms of advantages, many respondents talked about access to new treatments and that participation in clinical trials can contribute to the future health and well-being of family and community. Many patients indicated that clinical trials can potentially increase their chance of survival and that they would be willing to participate in them if they receive more information about benefits and side effects. Respondents also discussed the importance of Black/African Americans being part of trials to ensure that treatments are effective and decrease health inequities.

Disadvantages of participating in cancer clinical trials included economic, social, and psychological or emotional issues. In addition, some respondents were concerned about clinical trial procedures, side effects, and how the trial would benefit them (see Table 3).

**Table 3.** Perceived and experienced advantages and disadvantages of participation in cancer clinical trials

Issue	Illustrative quote
Advantages to participating in clinical trials at the personal, family, and community levels	*“For me, as I said earlier, (the trial) gave me access to a type of medication that wasn’t widely (available) at the time, and it worked for me.”* (Community)
	*“For me personally, it would be beneficial because I have a daughter. That would be my biggest concern because I have a daughter. I don't know. I guess I just feel differently about it now … . If I could participate in something that could help somebody else later down the line eases their mind or answer some questions, I mean, that would be a reason for me to participate.”* (Patient)
	*I want to say that I think clinical trials can be a good thing … . Whites have probably been involved in clinical trials, so we know how things affect them more vs Blacks because we have a lack of Black people being involved … . We don’t know maybe about how our ethnicity determines certain things when we respond to medicine.”* (Community
Economic disadvantages of participation in clinical trials	*“I can’t miss work if the trial isn’t going to benefit me as much. I understand the science and we need … . This is for the greater good. However, I have an immediate need, so maybe those things you can consider.”* (Community)
	*“Your question about the Black community and how our work may be a barrier, there’s many of us working in the service sector. So, they work during the day, they may not be able to get time off … so that may impact their ability to take time away from their paying jobs to do a trial. On the other hand, there may be some that are professionals who must work late hours like me.”* (Community)
Disadvantages in terms of family obligations	*“I have a family … when you put things in your body … you’re the patient. But if you are a mother, sister, wife, or husband, how will your changes affect that family? You know, when I think of clinical trials, I think of things like that.”* (Patient)
Disadvantages in terms of clinical trial procedures (eg, randomization)	*“I wanted to get out of it because they gave me a placebo. They ended up telling me that I was going to be in the placebo group. And then I had to do this biopsy and I’m like, ‘What is it going to benefit me?’ And it didn’t benefit me. But then I had to just pray on it and say, ‘Okay it’s going to benefit someone else.’ … So I went ahead and did it, but I didn’t want to do it because I didn’t see any benefit for it for me.”* (Community)

### Recruitment procedures and decision-making processes for cancer clinical trials

Recruitment into clinical trials requires that potential participants be given clear and comprehensive information about the trial requirements, procedures, and potential adverse events. During recruitment, potential participants need to feel confident that they are getting factual information and enough time to process that information so they can make informed decisions. Some individuals also want to hear from other patients who have participated in cancer trials or obtain a second medical opinion. In addition, decision making is not necessarily an individual act but may involve family and friends as well as spiritual, faith, and reflection as a part of the process (see Table 4).

**Table 4.** Recruitment procedures and decision-making processes in relation to cancer clinical trials

Issue	Illustrative quote
Confidence in hearing about clinical trial from a primary care physician	*“I would feel most comfortable hearing about (a clinical trial) from my primary physician because that’s who I have the relationship with. I see them an ongoing basis.” (*Patient)
Preference in obtaining information in a group format or from multiple members of a medical team	*“[P]resenting the information more in a group atmosphere, because when you’re in a group, just like we’re in a group right here, there’s so much you can learn from other people besides just yourself. Other people may ask a question that I wouldn’t ask.”* (Community)
	*“I would want to hear information and feedback from that entire community because I want to hear the perspective from all of them that’s involved in that clinical trial … . So not just one person.”* (Patient)
Confidence in information from cancer survivors	*“I think the (cancer) survivors too, people who are going through it. Because I suffered through three different breast cancers … . I would want to hear what they have to say and what they went through for the research or ideas.”* (Patient)
Family role in decision making	*“Okay. I would most definitely discuss it with my family. I want them to be as informed as I am on this whole journey. They need to know everything*.*”* (Patient)
	*“[M]y mother is now a 13-year breast cancer survivor. And so, she actually has walked the walk. Because prior to my diagnosis, I never had surgery or anything … . So I talked with my mom and she just pretty much walked me through everything from the diagnosis and the chemo treatments. And it gave me a sense of ease as to what was to come or to be expected.”* (Patient)
Importance of spiritual or religious support	*“I would like to talk to someone who can support me emotionally in making this decision … . I need someone to be there for me to encourage me spiritually and emotionally … a spouse, maybe family that I really trust.”* (Community)
	*“I would pray at about it and say, ‘Lord, you guide me this way and let me know if I should participate in something like this.’”* (Patient)

### Participant recommendations

Patients and community members were asked for their recommendations and suggestions to address the various barriers to Black/African American communities’ participation in cancer clinical trials. These recommendations spanned interventions within communities, clinical settings, and at the health-care system level (see Table 5). Many of these proposed recommendations addressed multiple issues and barriers raised during the focus group discussion.

**Table 5.** Recommendations to address barriers to Black/African American participation in cancer clinical trials

Type of intervention	Recommendations	Barrier categories			
		Systemic issues, racism, health disparities, and trust	Personal experiences with clinicians and health systems	Advantages and disadvantages of participation in cancer clinical trials	Recruitment procedures and decision-making processes
Community	Clinicians and pharmaceutical representatives participate in community-wide outreach into Black/African American communities to build trust.	X	X		
	Provide information about the importance of diversity in clinical trials to support effective treatments for Black/African American patients.	X		X	X
	Use of group discussions as a format to bring people together and share information and experiences.			X	X
Clinical	Establish positions for Black/African American community liaisons and cancer survivors to provide information and experiences related to cancer clinical trials.	X		X	X
	Train clinicians to be more proactive with Black/African American patients and communities about cancer prevention and early screening.	X	X		
	Clinicians need to recognize and promote the establishment of good relationships over the long term in terms of 2-way communication with patients in regular practice and during trial recruitment.		X		X
	Clinicians need to be trained to recognize patients who are overwhelmed when diagnosed with cancer.		X		X
	Information about procedures, time commitment, and costs needs to be shared during trial recruitment.			X	X
	Provide patients with sufficient time to ask questions and make an informed decision.	X	X	X	X
	Provide clinical trial information to family, partners, and friends who are part of the patient’s support network.			X	X
Systemic	Institutionalized outreach should be supported by health systems and conducted regularly to provide information about clinical trials and basic information about cancer prevention, screening, and treatment.	X			
	Establish a system for reporting clinical trial results to participants, enabling them to obtain information about trial outcomes and the implications of those outcomes for future cancer treatment and care in Black/African American communities.	X			
	Clinicians need to have more time built into the system to see patients, including during trial recruitment.		X		X
	Health systems need to provide equitable monetary incentives to address the clinical and the nonclinical impacts of the trial on patients’ lives.	X		X	X
	Administer clinical trials at multiple sites to increase accessibility to a broader population.			X	X
	Informed consent documents must be written in language that is simple to understand and clearly indicates the benefits, risks, time commitments, and direct and indirect cost to patients.	X	X	X	X

## Discussion

The current study elucidated the complex and multifactorial issues that influence the decision-making process of Black/African Americans about cancer clinical trial participation. Patients’ decisions to participate in clinical trials are influenced not only by the team of clinicians who manage their disease but also by the historical and current health-care experiences for themselves and their community. Every interaction with the health system, be it positive or negative, contributes to the decision-making process.

Patients and community members discussed the impact of a range of issues related to systemic racism and health disparities. Comments recalled historical and present-day experiences of harm and deliberate neglect by the government and the health-care system. They pointed to the lack of information about cancer prevention, screening, treatment, and clinical trials as a major barrier between the Black/African American community and the health-care system. The failure to communicate the outcomes of clinical trials for participants and the larger community was viewed as a lack of transparency. Respondents called for health-care systems to have a structured system to engage Black/African American communities over the long term to promote cancer prevention, screenings, and treatment.

The discomfort experienced during clinical trials, concerns about unequal treatment, and the potential harm from investigational medication have been recognized as a hindrance to trial participation in the Black/African American community.^28^ Comments affirmed that the emotional and mental distress linked to these apprehensions can influence people’s motivation to participate in clinical research. Recognizing and dealing with these issues are essential for trust building and promoting diverse involvement in trials.

The relationship between the clinician and the patient is especially important. When patients do not feel that their clinicians have an interest in their well-being, they are reluctant to participate in clinical trials. The quality of these relationships directly affects patient trust and confidence. When patients believe that their clinicians are committed to their well-being and care, they are more inclined to follow recommendations. Nurturing a positive and supportive connection between health-care professionals and patients is crucial for delivering effective health care and encouraging patient involvement in medical initiatives.

Effective communication is crucial to the development of trusting, strong relationships. Respondents emphasized that it is essential for clinicians to allocate sufficient time to address patients’ questions and concerns. It is crucial for patients to feel heard and for their concerns to be recognized as important components of their health-care experience. Health-care systems must also recognize the limitations that clinicians face in terms of having adequate time and resources during clinical visits to build and support trusting relationships with their patients in terms of cancer care and recruitment for clinical trials.

Beyond information from clinical experts, cancer survivors and community liaisons can be excellent resources to discuss and address patient concerns about cancer clinical trials. Community engagement is central to the development of acceptable and accessible interventions that address complex factors. Partnerships must be developed and maintained with trusted faith-based and community-based organizations to share clinical trial information and opportunities. These efforts to build trust and confidence can not only positively influence decisions regarding cancer clinical trial participation but also affect patient emotional state and mindfulness, which are integral to positive clinical trial outcomes. Health systems can further decrease disparities by increasing the representation of Black/African Americans within their health-care workforce and by increasing ethnic diversity and cultural inclusivity in the design and implementation of clinical trials.^29^ Studies have shown that many clinicians and clinical research professionals hold negative stereotypes of patients from minoritized communities, including nonadherence and low health literacy.^29^ Addressing these recommendations and making the health-care system more receptive to patients is beneficial not only to Blacks/African Americans but to all patients with cancer.

Training for research and clinical professionals can address bias and stereotypes about how nonadherence and low health literacy affect recruitment of individuals from minoritized communities. A comprehensive approach at the academic medical school, health system, health-care professional, patient care, and community levels is necessary to move forward in addressing clinical trial participation.^28-33^ Over the next 3 years, extension of the project (PAACT 2.0) will provide funding to continue selection and piloting of strategies to increase Black/African American participation in cancer clinical trials at HFCI. PAACT 2.0 has an expanded community advisory board and will continue to use approaches to maximize community engagement. Strategies will include programs within communities and clinics and at the health system level.

The current study used qualitative data to understand the breadth and depth of concerns among Black/African Americans regarding participation in cancer clinical trials and recommendations to address barriers. Future research should include a mixed-methods approach and focus on obtaining more generalizable data to increase understanding of the impact of the various barriers and facilitators on actual cancer clinical trial participation. The study was conducted within a single health system within the Detroit metropolitan area. Replication of the study in other Black/African American communities is necessary.

Although there were options in terms of participant recruitment, consenting, and participation, there could still be a bias in terms of decreasing participation among individuals and communities with reduced access to computers or the internet. In addition, selection of study participants by community members of the steering committee might have introduced selection bias, and participants may not fully represent the diversity within these communities. Future studies should also focus on differences in barriers and facilitators across and within Black communities (eg, Latin, African, Caribbean, and other ethnically diverse and marginalized communities).

Community-based participatory research is effective in bringing communities equitably to the table to identify and address issues that influence and affect participation in clinical trials. Community-based participatory research is an approach that fosters power sharing among community, academia, and health systems. Building trust between patients and the health-care system begins before patients walk into a clinic, and every interaction contributes to the worthiness of community and patient trust, which affects patients’ experiences, well-being, and health outcomes. It is possible and imperative for health systems to continue building trust by providing equitable opportunities to jointly identify and implement recommendations, address health disparities, and include the expertise and concerns of diverse communities.

## Supplementary Material

pkae119_Supplementary_Data

## Data Availability

Data will be made available at openICPSR (https://openicpsr.org/).

## References

[1] Giaquinto AN , Miller KD, Tossas KY, Winn RA, Jemal A, Siegel RL. Cancer statistics for African American/Black People 2022. CA Cancer J Clin. 2022;72:202-229. 10.3322/caac.21718 35143040

[2] Yedjou CG , Sims JN, Miele L, et al Health and racial disparity in breast cancer. Adv Exp Med Biol. 2019;1152:31-49. 10.1007/978-3-030-20301-6_3 31456178 PMC6941147

[3] Anstey EH , Shoemaker ML, Barrera CM, O'Neil ME, Verma AB, Holman DM. Breastfeeding and breast cancer risk reduction: implications for black mothers. Am J Prev Med. 2017;53:s40-s46. 10.1016/j.amepre.2017.04.024 28818244 PMC6069526

[4] McGinley KF , Tay KJ, Moul JW. Prostate cancer in men of African origin. Nat Rev Urol. 2016;13:99-107. 10.1038/nrurol.2015.298 26718455

[5] Bree KK , Hensley PJ, Pettaway CA. Germline mutations in African American men with prostate cancer: incidence, implications and diagnostic disparities. Urology. 2022;163:148-155. 10.1016/j.urology.2021.08.017 34453957

[6] Jones T , Lockhart JS, Mendelsohn-Victor KE, et al Use of cancer genetics services in African-American young breast cancer survivors. Am J Prev Med. 2016;51:427-436. 10.1016/j.amepre.2016.03.016 27117712

[7] Hoadley A , Bass SB, Chertock Y, et al The role of medical mistrust in concerns about tumor genomic profiling among black and African American cancer patients. Int J Environ Res Public Health. 2022;19. 10.3390/ijerph19052598 PMC8909390 35270290

[8] Somayaji D , Cloyes KG. Cancer fear and fatalism: how African American participants construct the role of research subject in relation to clinical cancer research. Cancer Nurs. 2015;38:133-144. 10.1097/NCC.0000000000000144 24945262

[9] Awidi M , Hadidi SA. Participation of Black Americans in cancer clinical trials: current challenges and proposed solutions. J Clin Oncol Oncol Pract. 2021;17:265-271. 10.1200/op.21.00001 PMC8258017 33974816

[10] Trantham LC , Carpenter WR, DiMartino LD, et al Perceptions of cancer clinical research among African American men in North Carolina. J Natl Med Assoc. 2015;107:33-41. 10.1016/s0027-9684(15)30007-9 26113749 PMC4477827

[11] Sprague Martinez L , Freeman ER, Winkfield KM. Perceptions of cancer care and clinical trials in the Black community: implications for care coordination between oncology and primary care teams. Oncologist. 2017;22:1094-1101. 10.1634/theoncologist.2017-0122 28706009 PMC5599206

[12] Eggly S , Hamel LM, Heath E, et al Partnering Around Cancer Clinical Trials (PACCT): study protocol for a randomized trial of a patient and physician communication intervention to increase minority accrual to prostate cancer clinical trials. BMC Cancer. 2017;17:807. 10.1186/s12885-017-3804-5 29197371 PMC5712160

[13] Dharni N , Armstrong D, Chung-Faye G, Wright AJ. Factors influencing participation in colorectal cancer screening-a qualitative study in an ethnic and socio-economically diverse inner city population. Health Expect. 2017;20:608-617. 10.1111/hex.12489 27550367 PMC5513014

[14] Linden HM , Reisch LM, Hart A Jr, et al Attitudes toward participation in breast cancer randomized clinical trials in the African American community: a focus group study. Cancer Nurs 2007;30:261-269. 10.1097/01.NCC.0000281732.02738.31 17666974 PMC3908682

[15] Hernandez ND , Durant R, Lisovicz N, et al African American cancer survivors' perspectives on cancer clinical trial participation in a safety-net hospital: considering the role of the social determinants of health. J Cancer Educ. 2022;37:1589-1597. 10.1007/s13187-021-01994-4 33728872 PMC8443686

[16] Frierson GM , Pinto BM, Denman DC, Leon PA, Jaffe AD. Bridging the Gap: Racial concordance as a strategy to increase African American participation in breast cancer research. J Health Psychol. 2019;24:1548-1561. 10.1177/1359105317740736 29172809

[17] Freedman TG. "Why don't they come to Pike Street and ask us"?: Black American women's health concerns. Soc Sci Med. 1998;47:941-947. 10.1016/s0277-9536(98)00167-1 9722113

[18] Michigan Cancer Consortium. https://www.michigancancer.org/Resources/ClinicalTrialsMCCOrg.html

[19] Israel BA, Schulz AJ, Parker EA, Becker AB. Critical issues in developing and following community-based participatory research principles. In: Minkler M, Wallerstein N, eds. *Community-Based Participatory Research for Health*. Jossey-Bass; 2008:47-62.

[20] Israel BA , Schulz AJ, Parker EA, Becker AB. Review of community-based research: assessing partnership approaches to improve public health. Annu Rev Public Health. 1998;19:173-202. 9611617 10.1146/annurev.publhealth.19.1.173

[21] Julian McFarlane S , Occa A, Peng W, Awonuga O, Morgan SE. Community-Based Participatory Research (CBPR) to enhance participation of racial/ethnic minorities in clinical trials: a 10-year systematic review. Health Commun. 2022;37:1075-1092. 10.1080/10410236.2021.1943978 34420460

[22] De las Nueces D , Hacker K, DiGirolamo A, Hicks LS. A systematic review of community-based participatory research to enhance clinical trials in racial and ethnic minority groups. Health Serv Res. 2012;47:1363-1386. 10.1111/j.1475-6773.2012.01386.x 22353031 PMC3418827

[23] Israel BA , Eng E, Schulz AJ, Parker EA. Methods for Community-Based Participatory Research for Health. Jossey-Bass; 2013.

[24] Wallerstein N , Duran B. Community-based participatory research contributions to intervention research: the intersection of science and practice to improve health equity. Am J Public Health. 2010;100(suppl 1):S40-S46. 10.2105/ajph.2009.184036 20147663 PMC2837458

[25] Center TDC-AUR. Detroit URCs CBPR principles. Accessed January 20, 2011. https://www.detroiturc.org/about-cbpr/community-based-participatory-research-principles

[26] Israel BA , Lichtenstein R, Lantz P, et al The Detroit Community-Academic Urban Research Center: development, implementation, and evaluation. J Public Health Manag Pract. 2001;7:1-19. 10.1097/00124784-200107050-00003 11680026

[27] Arring NM , Aduse-Poku L, Jiagge E, et al A scoping review of strategies to increase black enrollment and retention in cancer clinical trials. J Clin Oncol Oncol Pract. 2022;18:614-632. 10.1200/op.21.00863 35671413

[28] Fogel DB. Factors associated with clinical trials that fail and opportunities for improving the likelihood of success: A review. Contemp Clin Trials Commun. 2018;11:156-164. 10.1016/j.conctc.2018.08.001 30112460 PMC6092479

[29] Niranjan SJ , Martin MY, Fouad MN, et al Bias and stereotyping among research and clinical professionals: Perspectives on minority recruitment for oncology clinical trials. Cancer. 2020;126:1958-1968. 10.1002/cncr.32755 32147815

[30] Sadler GR , Gonzalez J, Mumman M, et al Adapting a program to inform African American and Hispanic American women about cancer clinical trials. J Cancer Educ. 2010;25:142-145. 10.1007/s13187-009-0032-y 20146043 PMC2878592

[31] Brown RF , Butow PN, Boyle F, Tattersall MH. Seeking informed consent to cancer clinical trials; evaluating the efficacy of doctor communication skills training. Psychooncology. 2007;16:507-516. 10.1002/pon.1095 16986176

[32] Anzai NE , Wieland J, Kasuya RT, Higuchi P, Cassel K. Medical school clinical trials educational intervention: impact on knowledge and attitudes. J Cancer Educ. 2023;38:1479-1485. 10.1007/s13187-023-02287-8 37170045 PMC10509125

[33] Nipp RD , Lee H, Gorton E, et al Addressing the financial burden of cancer clinical trial participation: longitudinal effects of an equity intervention. Oncologist. 2019;24:1048-1055. 10.1634/theoncologist.2019-0146 30988039 PMC6693715

