# Supplementary material for: Cancer clinical trial participation: a qualitative study of Black/African American communities’ and patient/survivors’ recommendations
Source: JNCI Cancer Spectr. 2024 Nov 25;9(1):pkae119. doi: 10.1093/jncics/pkae119 (PMC11781321; doi:10.1093/jncics/pkae119)
Supplement: pkae119_Supplementary_Data [file pkae119_supplementary_data.pdf]

Supplementary Materials for

Cancer Clinical Trial Participation: A Qualitative Study of Black/African American Communities' and Patient/Survivors' Recommendations

Linda Kaljee, Sylvester Antwi, Doreen Dankerlui, Donna Harris, Barbara Israel, Denise White-Perkins, Valerie Ofori Aboah, Livingstone Aduse-Poku, Harriet Larrious-Lartey, Barbara Brush, Chris Coombe, La'Toshia Patman, Nayomi Cawthorne, Sophia Chue, Zachary Rowe, Cassandra Mills, Fernando, Gwendolyn Daniels, Eleanor M. Walker, and Evelyn Jiagge*.

*Correspondence to: ejiagge1@hfhs.org

Supplementary Method

<u>INTERVIEW QUESTIONS (PATIENTS SURVIVORS)</u>

Knowledge and experiences with health care

We want to start with just a general discussion about your experiences with receiving health care.

1. Do you have a clinic/provider that you see regularly? How often do you go to that clinic/provider?
2. Can you tell me about positive experiences that you have had within health systems (e.g, clinics, hospitals)? Can you tell me about challenges or negative experiences you have had within health systems?
    a. **PROBES**: communication with HCPs; language barriers; asking questions and getting clear information/responses)
3. What factors/experiences have affected your feelings of trust in healthcare systems (hospitals/clinics)? How would these factors/experiences affect your decision about participating in a cancer treatment clinical trial?
4. What factors/experiences have affected your feelings of trust in medications/pharmaceuticals?
    a. **PROBES**: information heard from media/social media; positive/negative experiences with taking medications [self or others]

Experiences with cancer and cancer clinical research trials

We would now like to talk more specifically about some of your experiences with cancer and cancer clinical research trials.

1. *How were you first told about your cancer diagnosis? Who did you talk with/meet with?*
    a. ***PROBES:*** *Did you know the health care providers or other person who discussed your diagnosis with you?*
2. *What could have been done differently (better) in terms of learning about your diagnosis?*
3. *What experiences have you had with family members or friends who have had cancer?*
    a. ***PROBES:*** *experiences with health care system, with treatments, experience as a caregiver*
4. *How did those experiences impact you when you were given your diagnosis?*
5. Can you tell me what you have heard about clinical trials/research? OR: What comes to mind when you hear "cancer clinical trials/research"? Why do you think people do cancer research?
    a. **PROBES**: purposes and processes of CT, e.g., randomization?
6. Do you think it is important to include Black/African Americans in CCTs? Why? or Why not?
7. Tell me about you and cancer clinical research. Have you ever been asked to participate in a clinical trial? Have you ever participated in a clinical trial? Can you tell me more about those experiences?
    a. **[IF SOME PEOPLE ANSWER YES to #7]**
        i. What information was provided to you about the clinical trial? Was the information provided in a way that it was clear and easy to understand for someone not familiar with clinical trials? Who provided you with the information or where did you see recruitment information about the trial?
        i. Did you feel comfortable asking questions? Did you feel comfortable talking to the person who was providing you with information (why/why not)?
        ii. Did you feel that you had enough time to ask the questions you had about the trial?

        iii. What questions did you have for the person(s) providing you with information about the trial?

    b. **[IF SOME PEOPLE ANSWER NO to #7]** What information would you need to make a decision about participating in a clinical trial? What questions might you ask to learn more about the trial?

Clinical Trial Recruitment

We would now like to talk with you about different ways that people learn about clinical trials and how they feel about those processes.

1. Who would you feel most confident in hearing from about clinical trials and why?
    a. **PROBES**: physicians, nurses, 'patient navigators', survivors, other trial participants, media, trusted religious/spiritual community leaders
2. What formats and sources of information would you feel most confident in terms of learning about clinical trials and why?
    a. **PROBES:** one-on-one conversations, video recordings, written information, e.g., pamphlets

Perceived disadvantages and benefits of participating in clinical trials

We would like to know what you perceive are the disadvantages and benefits of participating in a clinical trial. This can be from your own experiences or experiences of family / friends. Or, you can just think about ways that being in a clinical trial would affect you.

1. What would be the primary benefits to you of participating in a clinical trial?
    a. **PROBES**: possibility of receiving new more effective treatments; supporting the development of more effective treatments for self and others; decreasing health disparities; learning more about clinical trials and development of treatments
2. What would be barriers or potential concerns for you about participation?
    a. **PROBES:** consent form issues, lack of control over treatment choices (randomization); possible unknown side effects; unknown treatment efficacy; possible effect of the treatment on other co-morbidities/ treatments for other co-morbidities; associated costs with participation; impact on time in relation to familial / social obligations, employment obligations, education

Decision-making processes in regard to participating in a clinical trial

This is our last section. We would like to learn more about how you made a decision to participate or not participate in a clinical trial. If you haven't been asked to participate in a trial, you can think about how you might make this decision.

1. Who **did you** discuss the clinical trial with as you were making your decision about participation? Can you talk about what were the key issues that you discussed? **AND/OR** Who **would** you discuss the clinical trial with **if** you were making a decision about participation and why?
    a. **PROBES**: spouse/partner, children/grandchildren; parents/grandparents; siblings; friends; co-workers; other health care providers, e.g., PCP
2. Did you seek spiritual / religious guidance? **AND/OR** Would you seek spiritual / religious guidance?
    a. **PROBES**: prayer, discuss with religious leader, discuss with other members of religious organization (e.g., church, mosque)
3. What were the key advantages or disadvantages related to participation in the trial that swayed your decision to participate or not participate?
4. What do you think could influence your decision to participate or not participate?

a. PROBES: varied examples of evidence-based successful interventions from the lit research i.e. handing out branded swag at community events, showing culturally tailored videos, etc.

5. In what way did the conversations with your health provider and/or those persons associated with the clinical trial affect your decision to participate or not participate?
6. Did you feel confident that you could make a good decision based on the information you had about the trial? Why or why not? Did you feel confident in the decision you made? Why or why not?
7. What could researchers/providers do to increase participation from Black/African Americans in CCTs?

Does anyone have any questions or comments for us? Are there any questions that we should have asked, but did not ask?

INTERVIEW QUESTIONS (COMMUNITY)

Knowledge and experiences with health care

We want to start with just a general discussion about your experiences with receiving health care.

1. Do you have a clinic/provider that you see regularly? How often do you go to that clinic/provider?
2. Can you tell me about positive experiences that you have had within health systems (e.g, clinics, hospitals)? Can you tell me about challenges or negative experiences you have had within health systems?
    a. **PROBES**: communication with HCPs; language barriers; asking questions and getting clear information/responses)
3. What factors/experiences have affected your feelings of trust in healthcare systems (hospital/clinics)? How would these factors/experiences affect your decision about participating in a cancer treatment clinical trial?
4. What factors/experiences have affected your feelings of trust in medications/pharmaceuticals?
    a. **PROBES**: information heard from media/social media; positive/negative experiences with taking medications [self or others]

Experiences with cancer and cancer clinical research trials

We would now like to talk more specifically about experiences with cancer and cancer clinical research trials.

1. What experiences have you had with family members or friends who have had cancer?
    a. **PROBES:** experiences with health care system, with treatments, experience as a caregiver
2. Can you tell me what you have heard about clinical trials?
    a. **PROBES**: purposes and processes of CT, e.g., randomization?

3. Have you ever been asked about participating in a clinical trial? Have you ever participated in a clinical trial? Can you tell me more about those experiences?
    a. **[IF SOME PEOPLE ANSWER YES to #3]**
        i. What information was provided to you about the clinical trial? Was the information provided in a way that it was clear and easy to understand for someone not familiar with clinical trials? Who provided you with the information or where did you see recruitment information about the trial?
        ii. Did you feel comfortable asking questions? Did you feel comfortable talking to the person who was providing you with information (why/why not)?
        iii. Did you feel that you had enough time to ask the questions you had about the trial?
        iv. What questions did you have for the person(s) providing you with information about the trial?

    b. **[IF SOME PEOPLE ANSWER NO to #3]** What information would you need to make a decision about participating in a clinical trial? What questions might you ask to learn more about the trial?

Clinical Trial Recruitment

We would now like to talk with you about different ways that people learn about clinical trials and how they feel about those processes.

1. Who would you feel most confident in hearing from about clinical trials?
    a. **PROBES**: physicians, nurses, 'patient navigators', survivors, other trial participants
2. What formats and sources of information would you feel most confident in terms of learning about clinical trials?
    a. **PROBES:** one-on-one conversations, video recordings, written information, e.g., pamphlets

Perceived disadvantages and benefits of participating in clinical trials

We would like to know what you perceive are the disadvantages and benefits of participating in a clinical trial. This can be from your own experiences or experiences of family / friends. Or, you can just think about ways that being in a clinical trial would affect you.

1. What are the primary benefits of participating in a clinical trial?
    a. **PROBES**: possibility of receiving new more effective treatments; supporting the development of more effective treatments for self and others; decreasing health disparities; learning more about clinical trials and development of treatments
2. What are the barriers or potential concerns about participation?
    a. **PROBES:** lack of control over treatment choices (randomization); possible unknown side effects; unknown treatment efficacy; possible effect of the treatment on other co-morbidities/ treatments for other co-morbidities; associated costs with participation; impact on time in relation to familial / social obligations, employment obligations, education

Decision-making processes in regard to participating in a clinical trial

This is our last section. We would like to learn more about how you made a decision to participate or not participate in a clinical trial. If you haven't been asked to participate in a trial, you can think about how you might make this decision.

1. Who **did you** discuss the clinical trial with as you were making your decision about participation? Can you talk about what were the key issues that you discussed? **AND/OR** Who **would** you discuss the clinical trial with **if** you were making a decision about participation?
    a. **PROBES**: spouse/partner, children/grandchildren; parents/grandparents; siblings; friends; co-workers; other health care providers, e.g., PCP
2. Did you seek spiritual / religious guidance? **AND/OR** Would you seek spiritual / religious guidance?
    a. **PROBES**: prayer, discuss with religious leader, discuss with other members of religious organization (e.g., church, mosque)
3. What were the key advantages or disadvantages related to participation in the trial that swayed your decision to participate or not participate?

4. In what way did the conversations with your health provider and/or those persons associated with the clinical trial affect your decision to participate or not participate?
5. Did you feel confident that you could make a good decision based on the information you had about the trial? Did you feel confident in the decision you made?

Does anyone have any questions or comments for us? Are there any questions that we should have asked, but did not ask?
